# Use of the "Two-Three Click" Protocol in Screw Stabilization of a Patient With Loosened Nuts and Dislocation of Rods - A Case Report

**DOI:** 10.7759/cureus.63373

**Published:** 2024-06-28

**Authors:** Plamen Penchev, Petar-Preslav Petrov, Kiril Ivanov, Ilko Ilyov, Remzi R Hyusein, Vladislav Velchev, Bogomil Iliev

**Affiliations:** 1 Medicine, Medical University of Plovdiv, Plovdiv, BGR; 2 Anatomy, Histology, and Embryology, Medical University of Plovdiv, Plovdiv, BGR; 3 Medicine, Medical University of Sofia, Sofia, BGR; 4 Neurosurgery, University Hospital Saint Marina, Varna, BGR; 5 Neurosurgery, Medical University of Varna, Varna, BGR

**Keywords:** posterior lumbar fusion, rod migration, inner development, screw stabilization malfunction, screw stabilization, case report, spinal stabilization, low back pain, nut loosening, screw nut

## Abstract

Vertebral fixation, utilizing titanium screws, is a highly prevalent technique employed to address spinal instability. Screw stabilization malfunction due to pedicle screw nuts loosening is rare. Under tightening the internal nut in the pedicle screw head may increase the likelihood of rod movement within the system resulting in severe pain when moving. Our goal is to raise the attention of surgeons when tightening the screws nuts of the screw stabilization because the consequences for the patient can be subsequent additional operations and complications.

This report describes a clinical case of a 40-year-old man who underwent three surgeries at different clinics several years ago for disc herniation at the L4-L5 level and screw stabilization at the same level. The patient presents to the neurosurgery clinic of Saint Marina University Hospital with a clinical manifestation of low back pain escalating with movement, with a pain intensity rating of six on the Visual Analogue Scale (VAS). From the CT scan, it was revealed a malfunction in the screw stabilization with loosening of the screw nuts and dislodgement of the rods. Screw stabilization was restored using intraoperative X-ray guidance and following the "two-three click" protocol. The patient was mobilized on the first day after surgery and discharged on the fifth day with neurological improvement (VAS=1). The patient was followed up for a period of six months, and no further complications were observed.

Surgeons must use caution while tightening the screw nuts, as not doing so may result in additional surgeries and complications for the patient in the future. The "two-three click" protocol for screw stabilization is an effective method for minimizing the issues associated with inner loosening and rod migration.

## Introduction

Pedicle screw fixation is a commonly employed technique in spine surgery. The device restricts the movement of the stabilized spine, enhances the rate at which fusion occurs, and is generally regarded as safe with a relatively low rate of complications [1,2]. However, in certain instances, the pedicle screw nut may develop, resulting in the migration of the rod and malfunctioning of the system [3,4]. There is a limited amount of research on this complication, with the majority of studies being in the form of individual case reports [2,5-7].

Our goal is to raise the attention of surgeons when tightening the screw nuts because the consequences for the patient can be subsequent additional operations and complications.

## Case presentation

Patient history

This report describes a clinical case of a 40-year-old patient who underwent three surgeries at different hospitals several years ago for disc herniation at the L4-L5 level and screw stabilization at the same level.

Clinical examination 

The patient presents to the neurosurgery clinic of Saint Marina University Hospital with clinical manifestation of low back pain increasing with movement with a pain intensity rating of six on the Visual Analogue Scale (VAS).

Imaging findings 

**Figure 1:**
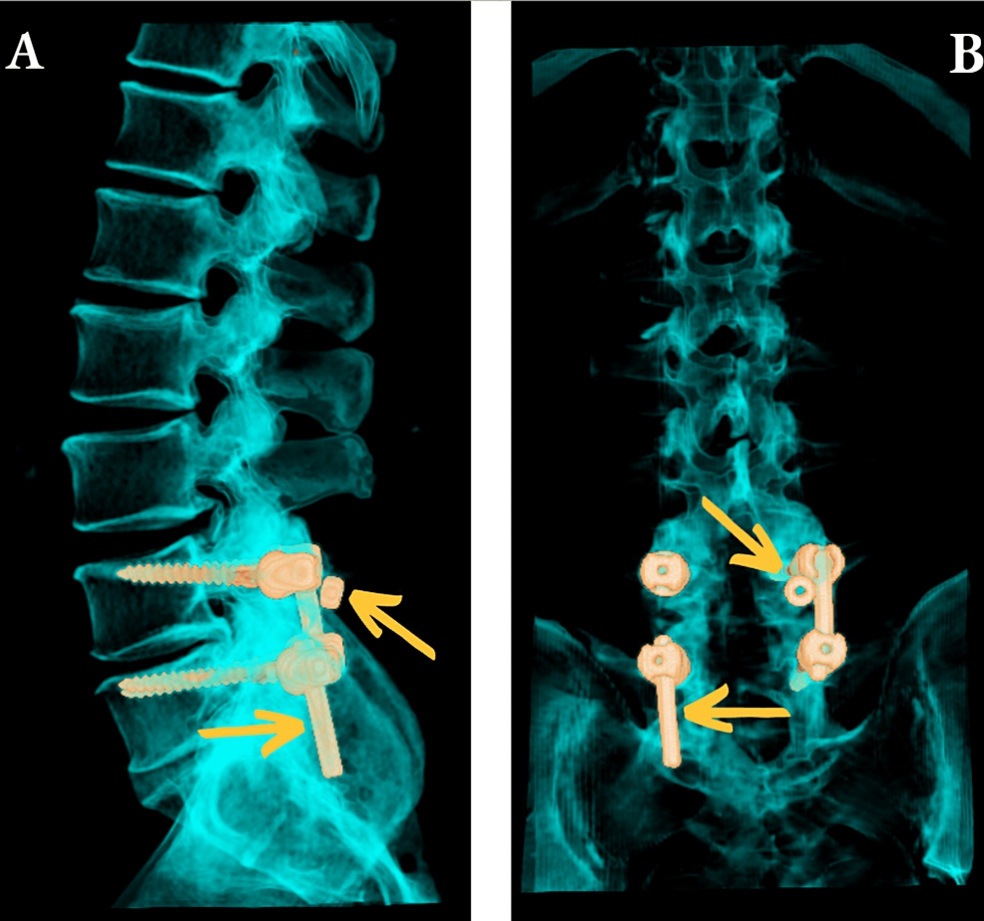
Preoperative 3D scan; the screw stabilization experienced a malfunction due to titanium rod migration and screw nut loosening A - sagittal plane, B - coronal plane

From a CT scan, it was revealed a malfunction in the screw stabilization with loosening of the nuts and movement of the rods (Figure 1).

Patient outcome

**Figure 2:**
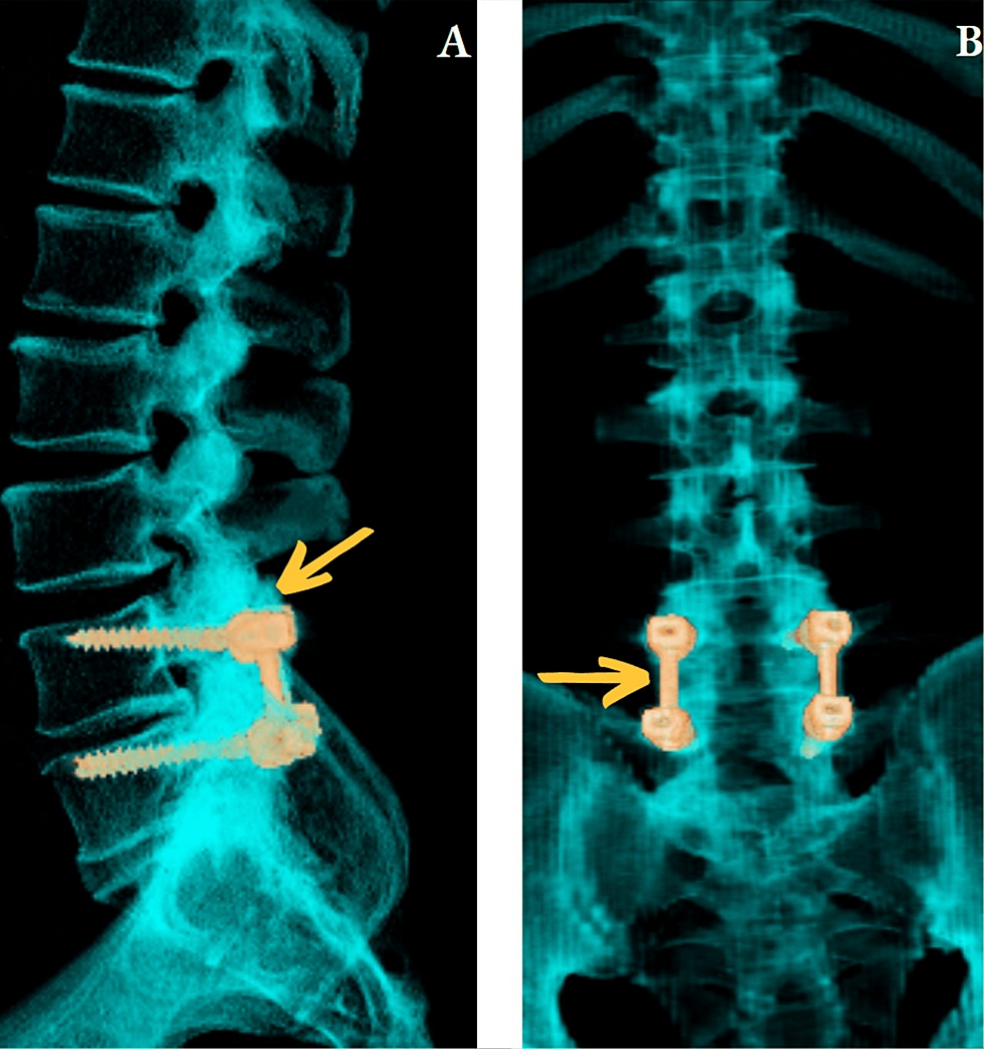
Postoperative 3D scan A - sagittal plane; B - coronal plane

Radiographically the screw stabilization was restored by retightening the nuts using standard tools; afterward, with a torque tool up to the "two-three clicks" protocol, all inner nuts were retightened (Figure 2). Surgery-related complications were not observed. The patient was verticalized on the first day after intervention and discharged on the fifth day with neurological improvement (VAS=1). The patient was followed up for a period of six months, and no further complications were observed.

## Discussion

Although transpedicular fixations offer certain benefits, they also include the potential dangers of nut loosening, screw loosening, screw breakage, screw/rod disconnection, pseudo-arthrosis, and nerve root injury, which can occur due to dural laceration and pedicle fracture during screw insertions. The occurrence of implant disassembly resulting from the loosening of the pedicle screw nut is extremely uncommon. If the inner nut is not adequately tightened into the pedicle screw head or the surgical team forgets to tighten the nuts additionally with the torque screwdriver, it can increase the likelihood of rod migration in the system [2-4]. Most of the time, only one of the inner components is developed. In our case, all four inners were developed.

We present a concise review of screw malfunction due to nut loosening cases reported in the literature (Table 1).

**Table 1:** A review of screw malfunction due to nut loosening cases reported in the literature

Author	Year	Patient data	Reason of screw malfunction	Clinical symptoms
Agrawal [2,6]	2014	65-year-old and 45-year-old male patients	Pedicle screw nut loosening	Back pain
					Kumar et al. [5]	2017	53-year-old female	Pedicle screw nut loosening	Back pain
Krishnan et al. [7]	2021	45-year-old and 58-year-old male patients	Pedicle screw nut loosening	Back pain
					Our case	2024	40-year-old male	Pedicle screw nut loosening	Back pain

According to Agarwal, Krishnan et al., and Kumar et al., the causes attributed to screw nut loosening include both inadequate tightening of the inner nut into the pedicle screw head, and incorrect alignment of the threads of both components before tightening can lead to nut loosening and dislodgment of the rod. The inner nut's threads align with the grooves on the pedicle screw, which can be accomplished by initially spinning the nut anticlockwise until a distinct "click" sound is heard [2,5-7].

**Figure 3:**
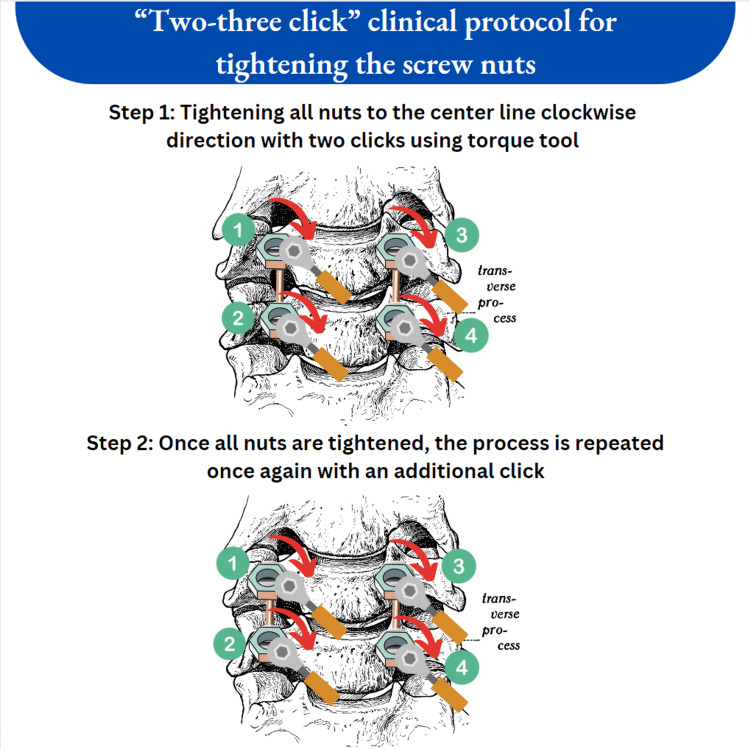
"Two-three click" clinical protocol for tightening the screw nuts This image was created with Canva by the author Plamen Penchev.

Regarding our patient, we speculate that during the procedure for screw placement, the surgical team either inserted the screw nuts crookedly or forgot to tighten them, additionally using a torque screwdriver. In order to avoid malfunction of the screw stabilization and loosening of the inners we recommend following the following steps. In the first step, to achieve optimal results, we recommend equally positioning the rods, being careful to prevent inserting muscles into the head of the screw prior to placing the inner part. In the second step, we recommend following our clinical protocol of "two-three clicks" when tightening the nuts using a torque tool (Figure 3). The protocol involves first tightening the nuts unilaterally to the left or right of the midline, then the opposite side in a clockwise direction with two clicks. Once all nuts are tightened, the process is repeated with an additional click. This approach eliminates the risk of forgetting an untightened inner. The protocol's effectiveness is demonstrated by the absence of any nut development or rod migration instances since its implementation in our neurosurgery clinic at Sveta Marina Hospital, Varna, Bulgaria. As a drawback is that a significant number of patients are no longer being followed up after a specific timeframe. Another drawback is that even though most stabilization systems share common components, certain systems, such as percutaneous screw stabilizations or dynamic stabilizations, require a particular type of internal locking, and in such cases, the protocol mentioned would not be suitable.

## Conclusions

Surgeons should be careful when tightening the screw nuts since this could result in later additional surgeries and complications for the patient. Patients who have had screw stabilization and experience worsening symptoms during movement should undergo a CT scan. The "two-three click" protocol in screw stabilization is a dependable method for minimizing the complications associated with nut loosening and rod migration. Аlthough in some individual cases, it would be inapplicable.
